# Using Mendelian inheritance errors as quality control criteria in whole genome sequencing data set

**DOI:** 10.1186/1753-6561-8-S1-S21

**Published:** 2014-06-17

**Authors:** Valentina V Pilipenko, Hua He, Brad G Kurowski, Eileen S Alexander, Xue Zhang, Lili Ding, Tesfaye B Mersha, Leah Kottyan, David W Fardo, Lisa J Martin

**Affiliations:** 1 Department of Pediatrics, Cincinnati Children's Hospital Medical Center, Cincinnati, OH 45229, USA; 2 Department of Pediatrics, University of Cincinnati College of Medicine, Cincinnati, OH 45229, USA; 3 Department of Environmental Health, University of Cincinnati College of Medicine, Cincinnati OH 45229, USA; 4 Department of Biostatistics, University of Kentucky, Lexington, KY 40356, USA

## Abstract

Although the technical and analytic complexity of whole genome sequencing is generally appreciated, best practices for data cleaning and quality control have not been defined. Family based data can be used to guide the standardization of specific quality control metrics in nonfamily based data. Given the low mutation rate, Mendelian inheritance errors are likely as a result of erroneous genotype calls. Thus, our goal was to identify the characteristics that determine Mendelian inheritance errors. To accomplish this, we used chromosome 3 whole genome sequencing family based data from the Genetic Analysis Workshop 18. Mendelian inheritance errors were provided as part of the GAW18 data set. Additionally, for binary variants we calculated Mendelian inheritance errors using PLINK. Based on our analysis, nonbinary single-nucleotide variants have an inherently high number of Mendelian inheritance errors. Furthermore, in binary variants, Mendelian inheritance errors are not randomly distributed. Indeed, we identified 3 Mendelian inheritance error peaks that were enriched with repetitive elements. However, these peaks can be lessened with the inclusion of a single filter from the sequencing file. In summary, we demonstrated that erroneous sequencing calls are nonrandomly distributed across the genome and quality control metrics can dramatically reduce the number of mendelian inheritance errors. Appropriate quality control will allow optimal use of genetic data to realize the full potential of whole genome sequencing.

## Background

Development of next-generation sequencing technologies has allowed for high-throughput genome sequencing. These advancements enable investigation of genetic association and linkage with high resolution; however, given the short read lengths in next-generation sequencing, error rates are much higher than traditional chip-based technologies [1]. Genotyping errors are a serious problem as an error rate as low as 1% to 2% can result in a false conclusion of linkage [2]. Several factors likely contribute to error rates, including sample preparation, sequencing platform variability, and sequence-specific characteristics. For example, certain areas of the genome are more likely to be associated with errors caused by structural and functional complexity, such as repetitive sequences. In population-based samples, these errors are often detected when follow-up Sanger sequencing fails to validate calls. However, with family based data, mendelian inheritance errors (MIEs) can help identify erroneous sequencing calls given that mutation occurs infrequently [3-5]. Although filters have been developed for whole genome sequencing (WGS) to identify regions of high complexity often associated with errors [1], there are no consensus guidelines for quality control procedures. Moreover, quantitative geneticists usually receive genotyping data in a very flexible and user-specified format called a VCF (variant call format) file. For instance, information related to quality controls (QCs) included in VCF files of the 1000 Genomes Project [6] differs from QC fields of VCF files used in the Genetic Analysis Workshop 18 (GAW18) that were generated by Complete Genomics (Complete Genomics, Mountain View, CA). Thus, our goal was to identify the characteristics that determine MIEs and explore QC information provided by VCF files. To accomplish this, we used GAW18 data of chromosome 3 WGS family based data. We found that MIEs are associated with repetitive DNA sequences and that QC variable such as SVM (support vector machine) can reduce MIEs.

## Methods

We analyzed sequence data of the human chromosome 3 obtained with DNA nanoarrays [7] generated by Complete Genomics. Variants listed in VCF file (VCFv4.0) were filtered to remove variants that didn't pass SVM (when SVM was less than zero; elements for SVM include allele balance, strand bias, fraction of bases with low quality, fraction of mendelian errors) or INDEL5 filters, and those that had more than 1 alternative nucleotide. Passing status for each variant was provided in the PASS column of the VCF file. We extracted MIEs from the INFO column. The VCF file included a field for MIEs. MIEs were identified as part of the Complete Genomics workflow using SimWalk2 [8,9]. We also calculated MIE for binary variants using PLINK [10]. Although SimWalk2 utilizes all of the available family data and considers recombination and haplotypes to estimate MIEs, PLINK estimates MIEs using nuclear families in a single-locus manner. Given the MIE detection method in PLINK, it is expected that PLINK will identify fewer errors than SimWalk2. However, given its speed and ease of use, PLINK is often the preferred method for MIE estimation in a large data set. Because the number of alleles affects probability of MIE detections [11], a small fraction of nonbinary variants (0.11%) was excluded from analysis to maintain homogeneous types of variants. The mean number of MIE per variant (MIE/variant) was calculated by dividing the total number of MIEs by the total number of variants. We used a Wald-Wolfowitz runs test implemented in the R package "lawstat" [12] to assess if MIEs (sum of MIEs per 1000 variants) were randomly distributed. Sums of MIEs per 1000 variants were plotted against their genomic positions. From this plot we detected MIE peaks. Identified MIE peaks were assessed for complexity using the RepeatMasker [13] track of the UCSC Genome Table Browser [14]. We further reduced MIEs using an SVM filter, listed among QC variables in the VCF file.

## Results

The uncleaned sequence data for the chromosome 3 is comprised of 1,757,461 variants. After removing variants that didn't pass SVM and INDEL5 filters, the number of variants were reduced by 8.5% to 1,607,227. The second filtering procedure removed variants that had more than 1 alternative nucleotide resulting in reduction of variants by 0.1% to 1,605,431. To examine the effect of second-stage filtering, we used the mean number of MIE per variant and number of variants with MIEs (Table 1). Importantly, the nonbinary variants (eg, variant with more than 1 alternative call) have a high rate of MIEs compared to biallelic variants. As such the remaining analyses include only binary variants.

**Table 1:** MIE content in data sets where variants were based on secondary filtering criteria

Data sets	No. of variants	No. of MIE	MIE/variant	Range of MIE per variant	No. of variants with MIEs
PASS	1,607,227	89,542	0.06	0-37	41,489 (2.6%)
Nonbinary	1,796	23,995	13.36	0-37	1,539 (85.7%)
Binary	1,605,431	65,547	0.041	0-22	39,950 (2.5%)

**Figure 1:**
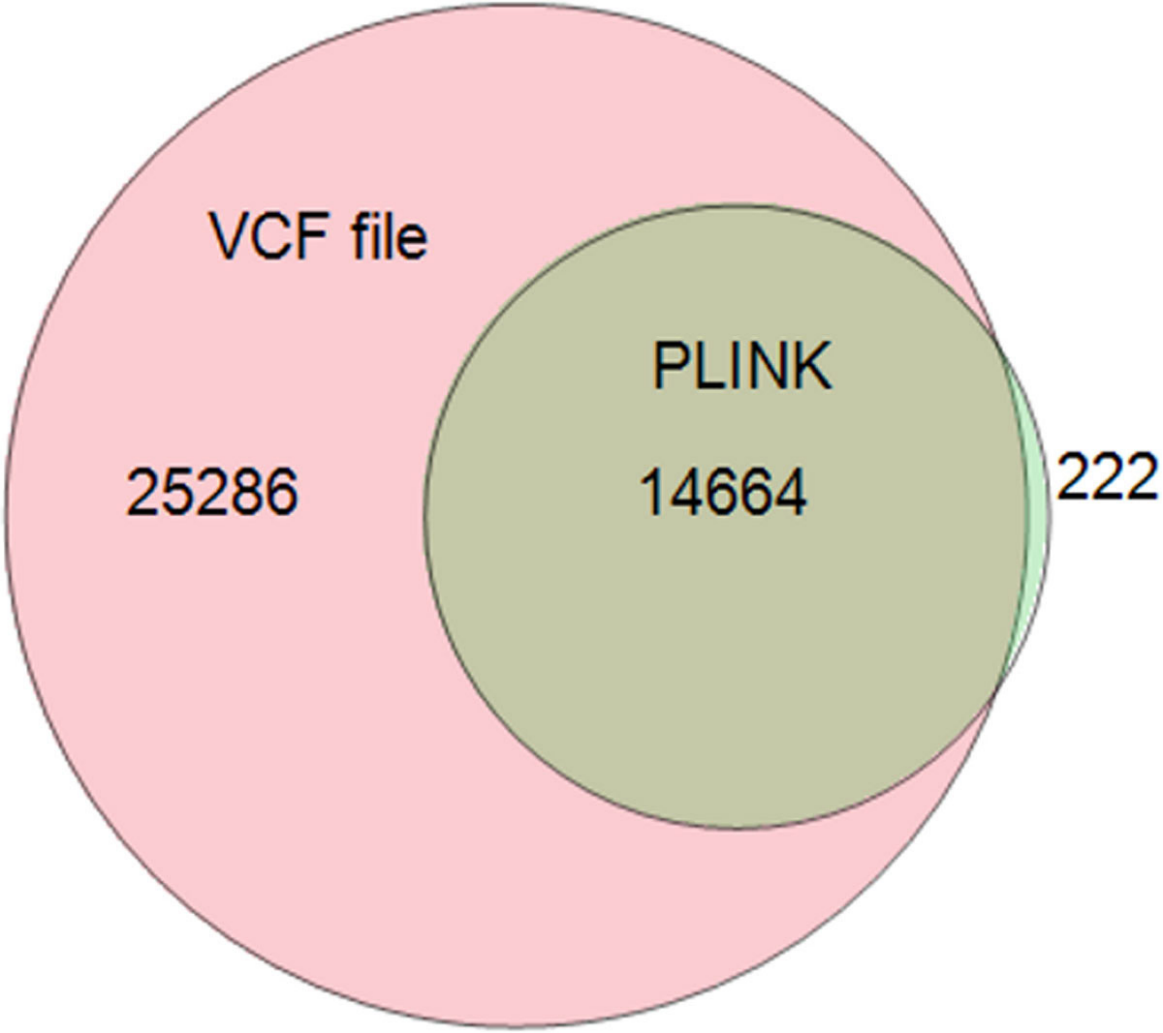
**Venn diagram of MIEs extracted from the VCF file and detected by PLINK**.

For binary variants we also calculated MIEs using PLINK. The number of variants with MIEs calculated by PLINK was significantly lower than the number of variants with MIE provided by the VCF file (14,886 and 39,950, respectively, p = 2.20E-16). Most of PLINK's MIEs were also flagged in the VCF file; only a small number of variants were identified by PLINK but not flagged in the VCF file (Figure 1).

**Figure 2:**
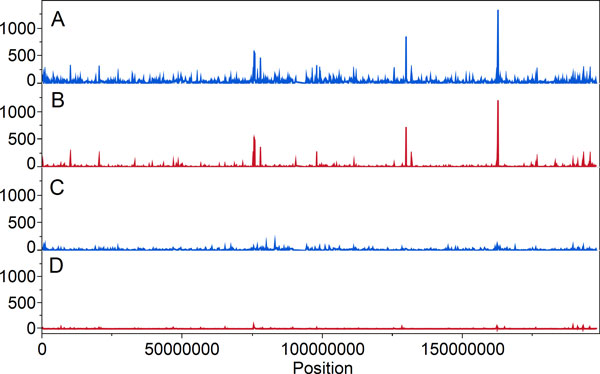
**Distribution of MIEs over the chromosome 3 in 1000 variant bins in the binary variants**. A. Peaks of MIEs where variants passed SVM and INDEL5 filters. B. Peaks of MIEs calculated using PLINK. C. Subsequent filtering where variants passed additional SVM filter with threshold >3.5. D. Subsequent filtering where variants passed additional SVM filter with threshold >3.5 and MIE calculation using PLINK.

**Figure 3:**
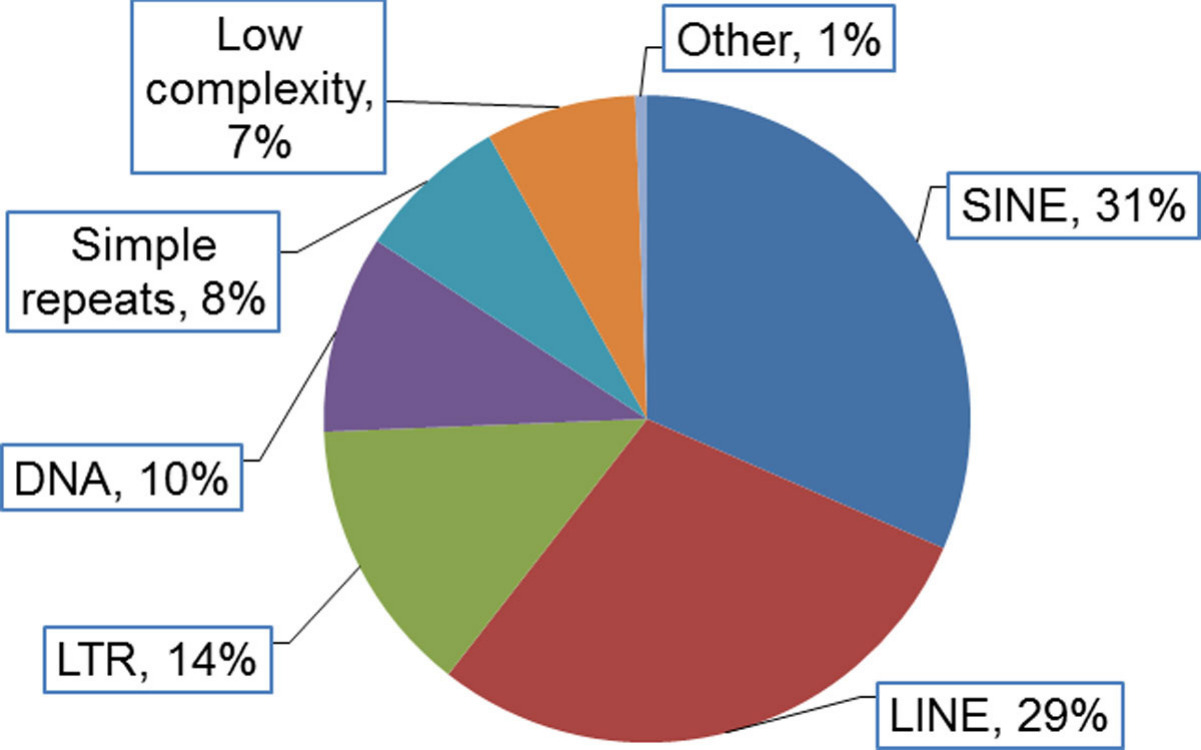
**Types of chromosome 3 repetitive elements**.

MIEs were nonrandomly distributed regardless of the MIE detection method (*p *value <2.2 × 10^−16^). We identified 3 MIE peaks (∑MIEs/1000 variants; Figure 2); Table 2 details the encoded transcripts located in these peaks. Overall, 49.26% of nucleotide bases in chromosome 3 comprise repetitive elements. Furthermore, MIE peaks were enriched for DNA repeats (68.11%, 55.96%, and 61.35% of repetitive sequence for peaks 1, 2, and 3, respectively). To further explore the relationship between MIE and presence of repetitive sequences, we determined total number of MIEs variants located in repetitive regions compared to variants without MIEs (Table 3). Variants with MIEs were more often located in areas with repetitive sequences, regardless of MIE detection method (*p *value <2.2 × 10^−16^). Most repetitive elements were SINEs (short interspersed transposable elements, 31.5%), LINEs (long interspersed transposable elements, 29.0%), and LTRs (long terminal repeats, 13.8%) (Figure 3).

**Table 2:** Locations of the MIE peaks

Peak	Location, hg19	Transcripts
1	75,536,587-75,821,588	*MIR1324, FRG2C, FLJ20518, LOC401074, MIR4273, ZNF717 *
2	129,767,883-129,837,072	*ALG1L2, FAM86HP*
3	162,441,039-162,675,707	Unknown

**Table 3:** Association of MIEs with areas in DNA sequences with repeats

Way of MIEs detection	Variants	Located in repetitive areas	Located in area free from repeats
From VCF file	with MIEs	26745	13205
	without MIEs	836713	728768

PLINK	with MIEs	10864	4022
	without MIEs	852594	737951

To reduce the MIE rate, we explored a number of parameters provided in the INFO column and found that an SVM parameter threshold of >3.5 was most effective at reducing error (Table 4). Additional SVM filtering results of the runs test demonstrated that MIE distribution remained nonrandom regardless of MIE detection method (*p *value <3.6 × 10^−7^).

**Table 4:** Reduction in MIE rate after employing SVM filter with different thresholds

Data sets	No. of variants	No. of MIEs	Mean no. of errors per variant	Range of MIEs per variant	No. of variants with MIEs
SVM >2	1462201	40816	0.03	0-17	25,706(1.76%)
SVM >2.5	1407230	33775	0.02	0-17	22,094(1.57%)
SVM >3	1334454	26530	0.02	0-17	18,324(1.37%)
SVM >3.5	1236087	19662	0.02	0-10	14,511(1.17%)
SVM >4	1107983	13438	0.01	0-7	10,629(0.96%)

## Discussion

Through examination of MIEs, we demonstrated that MIEs were nonrandomly distributed over human chromosome 3, with several peaks enriched for errors. These peaks were localized in regions of repetitive sequence. Importantly, we found that using an SVM filter reduced MIEs.

The number of MIEs from PLINK was significantly lower than the number of MIEs flagged by Complete Genomics. These differences may be because PLINK calculates MIEs by dissecting a large pedigrees into nuclear families as compared to the MIEs from Complete Genomics which was based on extended families. However, distributions of MIEs exhibited a similar pattern between the two methods. Specifically, both methods identified 1 major and 2 moderate peaks occurring in a similar location. However, after SVM application of SimWalk2, there were more minor MIE peaks (see Figure 2C) than MIEs from PLINK (see Figure 2D), possibly as a result of the smaller number of variants with MIEs detected by PLINK.

Although Complete Genomics has a reported accuracy of greater than 99.999% [15], this accuracy is achieved after substantial data cleaning. Indeed, the uncleaned sequence data for chromosome 3 includes 1,607,227 variants passing SVM and INDEL5 filters. However, the cleaned data set contained 1,215,399 variants. The proprietary nature of the workflow doesn't describe filtering procedures used; removal of MIEs (41,489) from the binary data set would not result in the actual reduction seen between the unclean and clean data set. Common parameters such as depth of coverage were not provided. Instead, we found that variants with more than 1 alternative nucleotide call have extremely high MIEs and should be considered suspect.

Using the binary set for analysis, we demonstrated that MIE peaks corresponded with regions of sequence complexity. Furthermore, we identified an SVM filter that significantly reduced MIEs. Although previous work on Complete Genomics data has suggested various filters to improve QC [16], an SVM filter was not applied. As an SVM work flow for genotype calling has been used for the 1000 Genomes Project [17] and the Exome Project [18], identifying SVM filters that improve data quality is important. The user-specific nature of VCF files highlights an important point that QC metrics may differ based on workflow. Thus, future studies will need to explore this important issue.

## Conclusions

In summary, examination of the areas with increased MIEs revealed that these areas were made up of repetitive sequence. Given that there is no consensus on filters to improve QC, identification of features associated with sequencing error will improve data quality and will allow optimal use of genetic data to realize the full potential of WGS studies.

## Competing interests

The authors declare that they have no competing interests.

## Authors' contributions

VP performed the statistical analysis and drafted the manuscript. LJM conceived the design of the statistical analysis. HH helped with statistical analysis. BGK, ESA, TMB, DWF, HH, XZ, LD, LK and LJM helped with the writing of the manuscript. All authors read and approved the final manuscript.
